# Cross-species validation of cell cycle arrest markers for acute kidney injury in the rat during sepsis

**DOI:** 10.1186/s40635-016-0086-1

**Published:** 2016-05-31

**Authors:** Zhi-Yong Peng, Feihu Zhou, John A. Kellum

**Affiliations:** Department of Critical Care Medicine, The Center for Critical Care Nephrology, CRISMA (Clinical Research, Investigation, and Systems Modeling of Acute Illness) Center, University of Pittsburgh School of Medicine, 604 Scaife Hall, 3550 Terrace Street, Pittsburgh, PA 15261 USA; Department of Critical Care Medicine, Zhongnan Hospital of Wuhan University School of medicine, Wuhan, 630071 China; Department of Critical Care Medicine, Chinese People’s Liberation Army General Hospital, Beijing, China

**Keywords:** Acute kidney injury, Sepsis, Tissue inhibitor of metalloproteinases (TIMP)-2, Insulin-like growth factor binding protein 7 (IGFBP7), Cell cycle arrest, Biomarkers

## Abstract

**Background:**

The recent discovery of cell cycle arrest biomarkers, tissue inhibitor of metalloproteinases (TIMP)-2 and insulin-like growth factor binding protein 7 (IGFBP7), has led to a newly available clinical test for acute kidney injury. The performance of these markers in preclinical studies has not been established. Therefore, we sought to evaluate the performance of TIMP-2 and IGFBP7 in rats undergoing cecal ligation and puncture.

**Methods:**

In this secondary analysis, we analyzed banked urine samples from 60 Sprague-Dawley rats undergoing cecal ligation and puncture (CLP). Samples were obtained from baseline, 18 h after CLP, at the end of fluid resuscitation (22 h after CLP), and again 24 h later. We measured TIMP-2 and IGFBP7 and compared the results to acute kidney injury by RIFLE criteria for creatinine using area under the receiver operating characteristic curve (AUC). The primary endpoint was moderate-to-severe acute kidney injury (AKI) (I or F criteria), and the primary time point was immediately after fluid resuscitation. Secondary outcomes included mortality and comparisons with other biomarkers: cystatin C and neutrophil gelatinase-associated lipocalin (NGAL) in both urine and plasma.

**Results:**

After fluid resuscitation, urine [TIMP-2] and [IGFBP7] were significantly higher in animals developing moderate-to-severe AKI (*p* = 0.002 and *p* = 0.01). AUC of [TIMP-2]·[IGFBP7] for AKI was 0.89 (95 % CI 0.80–0.98). By contrast, the next best AUC was seen with plasma cystatin C (0.78; 95 % CI 0.65–0.90). [TIMP-2]·[IGFBP7] also predicted mortality (AUC 0.69; 95 % CI 0.53–0.85).

**Conclusions:**

In this experimental model of sepsis in the rat, cell cycle arrest biomarkers TIMP-2 and IGFBP7 are valid predictors of acute kidney injury.

## Background

Recently, two novel urinary biomarkers, tissue inhibitor of metalloproteinases-2 (TIMP-2) and insulin-like growth factor-binding protein 7 (IGFBP7), were validated for predicting moderate-to-severe acute kidney injury (AKI) (stages 2 and 3 by Kidney Disease Improving Global Outcome (KDIGO) [1] criteria) in critically ill patients [2, 3]. Both TIMP-2 and IGFBP7 are markers of G1 cell-cycle arrest, which prevents cells from dividing when potentially injured [4], and both appear to respond to a wide variety of cellular stressors including inflammation, ischemia, oxidative stress, drugs, and toxins [5–7]. Furthermore, both molecules also act as “alarm” proteins exerting paracrine effects on adjacent cells [8]. Novel AKI biomarkers may play important roles clinically [9], but they also have great potential to transform clinical trials [10, 11]. As such, it is important to understand how these markers perform in preclinical models of AKI. Sepsis is the most common cause of AKI in critically ill patients [12, 13], and cecal ligation and puncture (CLP) in small animals is a commonly used preclinical model.

Thus, we sought to evaluate the performance of [TIMP-2]·[IGFBP7] in an rat model of sepsis. In order to be consistent with the human trials that were used to discover and validate these biomarkers [2, 3], we used moderate-to-severe AKI (equivalent to KDIGO stages 2–3 or RIFLE I-F) as the primary outcome. We assessed biomarker performance at various time points, but we used the immediate post-resuscitation time point as primary because it most closely matches the time when patients were enrolled in these trials.

## Methods

For this analysis, we used banked samples from 60 animals used in a prior laboratory experiment involving rats subject to cecal ligation and puncture and randomized to resuscitation with two different crystalloids: 0.9 % saline and plasmalyte. Complete details of the parent study have been published [14]. Briefly, 24- to 28-week-old (weight 400–600 g) male, Sprague-Dawley rats were anesthetized with intraperitoneal injection of pentobarbital sodium (50 mg/kg) and subjected to CLP with a predetermined 25 % ligated length of cecum and 18-gauge needle: two punctures inferior to the ileocecal valve. Eighteen hours after CLP animals were re-anesthetized, vascular cannula were placed and animals received 10 ml/kg fluid resuscitation in the first hour with either 0.9 % saline or plasmalyte. For the next 3 h, animals received the same fluid at 5 ml/kg/h. Animals were observed for survival over an additional 24 h. Blood (1 ml) was drawn from the arterial line, and urine (1–2 ml) was taken from the bladder at 0 (baseline), 18 (before fluid resuscitation), 22 (end of fluid resuscitation), and 24 h after fluid resuscitation (46 h after CLP). Isolated plasma and urine was kept at −80 °C for subsequent neutrophil gelatinase-associated lipocalin (NGAL), cystatin C, and creatinine (Cr) measurements and the remainder stored.

Plasma NGAL and urine NGAL were determined using ELISA (BioPorto Diagnostics, Gentofte, Denmark). Plasma creatinine was detected with a creatinine enzymatic assay kit (BioVision Technologies, Mountain View, CA). Plasma cystatin C and urine cystatin C were measured by ELISA (BioVendor LLC, Candler, NC). Frozen urine samples were used for measurement of TIMP-2 and IGFBP7 by ELISA. The ELISA for TIMP-2 was made using capture and detect antibodies from R&D Systems (Minneapolis, MN) and the ELISA for IGFBP7 using capture and detect antibodies from Thermo Fisher Scientific (Waltham, MA) and R&D Systems, respectively. TIMP-2 and IGFBP7 protein from Abcam (Cambridge, UK) and U-Protein Express (Utrecht, Netherlands), respectively, were used for calibration. The calibration range for both assays was 0.0156 to 2 μg/mL.

We determined the severity of AKI using the serum creatinine portion of the RIFLE criteria [15], which classified risk (R), injury (I), and failure (F), on the basis of maximum creatinine increase of 150, 200, and 300 %, respectively, in the 2 days following CLP. For the primary analysis, we compared I and F to R and no AKI. Biomarker data are expressed as means ± standard error (SE). Student’s *t* tests were applied to compare means. Biomarker concentrations were compared across the first three time points using repeated measures ANOVA with Huynh-Feldt correction. The fourth time point was not included because only 19 rats had both TIMP-2 and IGFBP7 results for all four time points. Dunnett’s test was used for pairwise comparisons with the baseline time point as the control. We calculated area under the receiver operator characteristic curve (AUC) and 95 % confidence intervals [16]. Analyse-it (Analyse-it Software, Ltd., UK) and XLSTAT (Addinsoft, Paris, France) software were used for statistical analysis. A two-sided *P* < 0.05 was considered statistically significant.

## Results

### TIMP-2, IGFBP7, and AKI

**Fig. 1:**
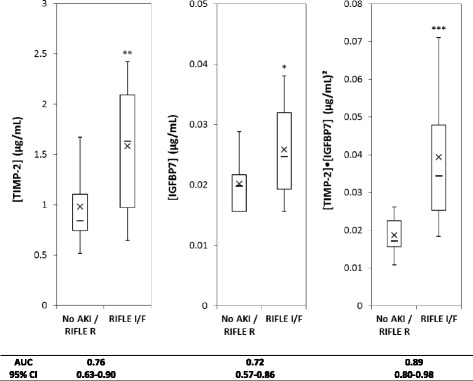
Urinary [TIMP-2], [IGFPB7], or [TIMP-2]·[IGFPB7] levels from animals with no AKI (or only RIFLE R) (*n* = 18) compared to RIFLE I or F (*n* = 30). *Box and whiskers* show interquartile range and 5th to 95th percentiles, respectively. *Horizontal dash* shows the median, and *X* shows the mean. *P = 0.01, **P = 0.002, ***P < 0.001

**Fig. 2:**
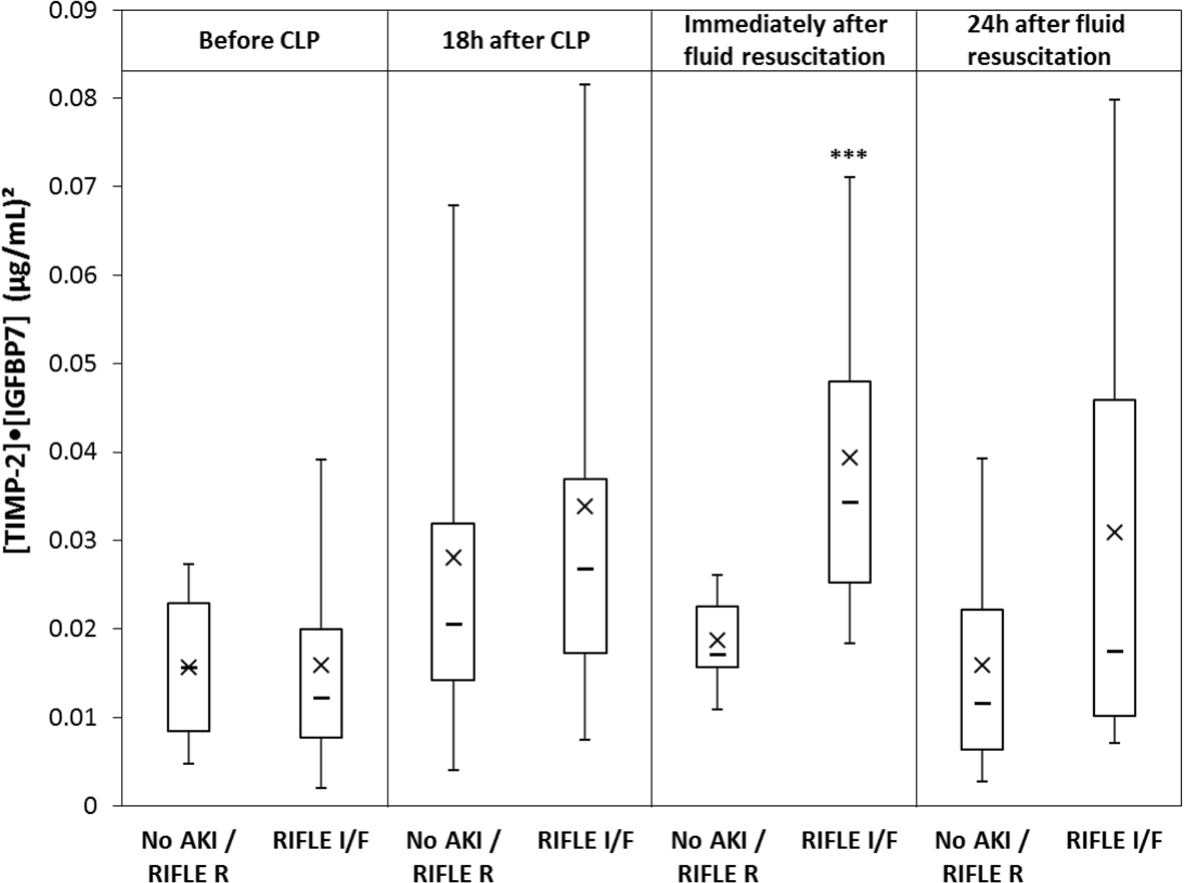
Box-and-whisker plots for [TIMP-2]·[IGFPB7] at various time points comparing no AKI or RIFLE R versus RIFLE I or F. ***p < 0.001 for immediately after fluid resuscitation

Samples were available at 18 and 22 h (right before and after fluid resuscitation) from 48 animals. Of these, 30 developed RIFLE I/F (18 I and 12 F) and 18 either had no AKI (8) or R (10). For animals developing AKI, creatinine peaked most often, at the 48-h time point. Immediately after fluid resuscitation, 22 h after CLP, urine concentrations of both TIMP-2 and IGFBP7 were greater in animals who ultimately manifested RIFLE I/F compared to those who did not (Fig. 1). The AUC for [TIMP-2]·[IGFBP7] for AKI was 0.89 (95 % CI 0.80–0.98). Interestingly, the AUCs for individual markers were significantly (*p* < 0.05) lower than the composite (0.76 and 0.72 for TIMP-2 and IGFBP7, respectively). There were no significant differences between the performance of TIMP-2 and IGFBP7 in animals receiving saline or plasmalyte. Mean [TIMP-2]·[IGFBP7] at 18 and 22 h were similar for animals receiving plasmaltye compared to saline (at 18 h 0.034 (95 % CI 0.023–0.046) vs. 0.028 (μg/ml)^2^ (95 % CI 0.017–0.039), *p* = 0.43 or at 22 h 0.034 (95 % CI 0.025–0.042) vs. 0.029 (μg/ml)^2^ (95 % CI 0.022–0.037), *p* = 0.43). However, fewer animals developed the endpoint in the plasmalyte group as reported previously [14]. At baseline, [TIMP-2]·[IGFBP7] results were not different between those animals who ultimately developed AKI compared to those who did not (Fig. 2). Both TIMP-2 and IGFBP7 increased 18 h after CLP compared to baseline (*p* = 0.01 and *p* < 0.0001, respectively). However, neither [TIMP-2]·[IGFBP7] (Fig. 2) nor individual markers (data not shown) discriminated well for AKI at the 18-h (pre-resuscitation) time point.

### Comparison with other urine biomarkers

Performances for various AKI biomarkers are shown in Table 1. The same time point (immediately after resuscitation) and same outcome, RIFLE I/F, were used for all comparisons. The AUC for [TIMP-2]·[IGFBP7] was significantly greater than for urine NGAL (0.89 vs. 0.71, *P* < 0.05) or plasma NGAL (0.89 vs. 0.62, *P* < 0.05; Table 1).

**Table 1:** Comparison of biomarkers for predicting RIFLE I/F

Biomarker	No AKI	AKI	*p* value	AUC
[TIMP-2]·[IGFBP7] (μg^2^/mL^2^)	0.019 ± 0.001	0.039 ± 0.003	<0.0001	0.89 (0.80–0.98)
Urine NGAL (IU/mL)	2157.6 ± 102.4	2472.8 ± 123	0.04	0.71 (0.63–0.80)
Plasma NGAL (IU/mL)	2143.3 ± 29.6	2077 ± 44.5	0.21	0.62 (0.46–0.79)
Urine cystatin C (IU/mL)	1609.8 ± 284.6	2572.8 ± 353.6	0.03	0.75 (0.56–0.93)
Plasma cystatin C (IU/mL)	1027.3 ± 185.8	2344.9 ± 462.5	<0.01	0.78 (0.65–0.90)

All comparisons are for the post-resuscitation (primary) time point. Shown are means ± standard error and areas under the receiver operating characteristic curves (AUC) with 95 % confidence intervals. *P* values are for two-sided *t* test

### Mortality

**Fig. 3:**
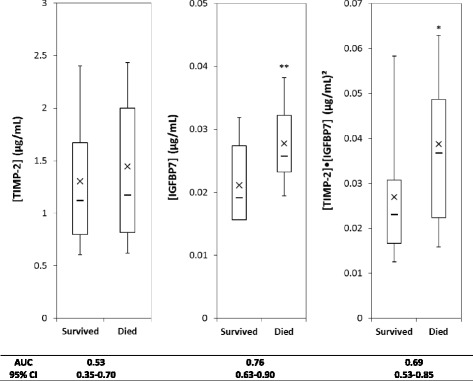
Urinary [TIMP-2], [IGFPB7], or [TIMP-2]·[IGFPB7] levels from animals that survived (*n* = 29) compared to those that died (*n* = 19). *Box and whiskers* show interquartile range and 5th to 95th percentiles, respectively. *Horizontal dash* shows the median, and *X* shows the mean. *P = 0.03, **P = 0.002

Early mortality (prior to day 2 after CLP) was observed in 19 animals. All deaths exhibited AKI. Immediately after fluid resuscitation, 22 h after CLP, urine concentrations of IGFBP7 (*P* = 0.002) but not TIMP-2 (*P* = 0.48) were greater in animals who died prior to day 2 compared to those who did not (Fig. 3). The AUC for [IGFBP7] alone was 0.76 (95 % CI 0.63–0.90) whereas the AUC for [TIMP-2]·[IGFBP7] for AKI was 0.69 (95 % CI 0.53–0.85).

## Discussion

Prior animal studies have examined TIMP-2 in relation to AKI [17, 18], and several studies have examined IGF binding proteins in renal disease [19] but none have directly determined the performance of [TIMP-2]·[IGFBP7] for the prediction of RIFLE I/F AKI in an experimental model. Thus, to our knowledge, this is the first cross-species validation of [TIMP-2]·[IGFBP7] for AKI and establishes the test as a viable tool in the preclinical space. This is important because clinical trials that use [TIMP-2]·[IGFBP7] for enrichment will benefit from preclinical data that closely matches the trial design. For this reason, we designed this laboratory investigation to simulate the clinical situation as much as possible. We used moderate-to-severe AKI (RIFLE I/F = KDIGO 2/3) as the endpoint, and we tested biomarkers at a time that closely mimics enrolment in clinical trials as well as clinical use in general.

There were several unexpected results of our investigation. First, earlier measurement of [TIMP-2]·[IGFBP7] (at 18 h after CLP and prior to fluids), while demonstrating an increase compared to baseline, was not predictive of RIFLE I/F AKI. We speculate that early increases in TIMP-2 and IGFBP7 might have been protective for some animals while sustained elevations were consistently associated with development of AKI. This result may help explain recent findings in humans where early release of TIMP-2 and IGFBP7 was associated with the protection from AKI in the setting of remote ischemic preconditioning and subsequent cardiac surgery [11]. The mechanisms responsible for this effect are still unclear, but we have proposed that limb ischemia releases damage-associated molecular patterns that signal the kidney to release TIMP-2 and IGFBP7 as part of an “alarm” that protects cells from subsequent injury [20]. Since temporary cell cycle arrest is a protective mechanism, it is reasonable to expect that under certain circumstances the mechanism works and release of TIMP-2 and IGFBP7 is not followed by AKI [9]. We speculate that pre-resuscitation levels may identify “renal stress” but not necessarily predict the effectiveness of the resuscitation. Whereas post-resuscitation levels may be more predictive of the pathologic (as opposed to adaptive) state. Alternatively, animals at pre-resuscitation may in fact already have renal injury but it may still be reversible. For animals that responded to resuscitation, they rapidly resolved this injury and no longer exhibited biomarker signatures nor did they manifest clinical AKI. Further study will be necessary to test these hypotheses. However, the finding does have implications for clinical trials and clinical care. For example, the test may have less utility in the pre-hospital arena. Second, it may point to a need to retest patients if clinical evaluation suggests that they were not resuscitated on initial testing.

Another unexpected finding was the wide separation between the AUCs for TIMP-2 and IGFBP7 compared to [TIMP-2]·[IGFBP7] for AKI. These differences were much greater than what was observed in humans [2]. Interestingly, in the clinical study by Kashani et al., TIMP-2 had a superior AUC compared to IGFBP7 in patients with sepsis, whereas the opposite was seen in surgical patients [2]. We observed better performance for TIMP-2 in this experimental model of sepsis (Fig. 1). These findings suggest that sepsis-induced AKI may have a unique underlying pathobiology.

Finally, we observed that while TIMP-2 was a better performing marker for AKI, it was a very poor predictor of mortality and IGFBP7 was better for this outcome (AUC 0.76 vs. 0.53); though not quite significant (*p* = 0.06) (Fig. 3). This uncoupling of predictors of AKI and mortality was unexpected and may warrant further investigation.

Our study also showed that urine [TIMP-2]·[IGFBP7] was superior to NGAL. NGAL derivation and validation studies were primarily performed in ischemic or nephrotoxic AKI, while investigations of NGAL in septic AKI have demonstrated variable results [21]. NGAL is known to be released by activated neutrophils and appears to be elevated in sepsis, which may complicate its diagnostic value for AKI in patients with sepsis. Our study also used RIFLE I/F AKI criteria (creatinine only) as the primary endpoint so as to more closely model the clinical scenario in humans. Of note, we have previously shown that in antibiotic-treated animals using this exact model, 1-week mortality for RIFLE-F was 36.4 % [22]. This is remarkably similar to 40.3 % crude hospital mortality reported for critically ill patients with KDIGO stage 3 AKI (using creatinine alone) [23] or the 32.4 % covariate-adjusted hospital mortality for KDIGO stage 3 AKI (using both creatinine and urine output) [13].

Our study has important limitations. As a secondary analysis, we were limited in what time points were available. It would have been useful, for example, to have measurements every 1–2 h. However, it is unlikely that we could have obtained this frequency of monitoring in our rats even if we would have tried. Secondly, we chose a relatively high severity model with substantial early mortality (40 % by day 2). It is not known whether a less severe model would have resulted in similar or different results. Finally, our model involved fluid resuscitation with saline in half of the animals, and indeed, the majority of animals reaching the AKI endpoint received saline. Therefore, the exposure is “sepsis plus saline” rather than sepsis alone. However, this mirrors the clinical reality in much of the world especially North America where sepsis patients are invariably resuscitated with saline [24].

## Conclusions

In conclusion, we have validated the [TIMP-2]·[IGFBP7] test in an experimental model of sepsis-associated AKI using CLP. Our results establish the test in the preclinical space.
